# Oral Health-Related Quality of Life among Croatian University Students

**DOI:** 10.3390/ijerph18126483

**Published:** 2021-06-16

**Authors:** Zvonimir Uzarevic, Ana Bulj

**Affiliations:** Faculty of Education, University of Osijek, 31000 Osijek, Croatia; abulj@foozos.hr

**Keywords:** oral health-related quality of life (OHRQoL), oral health impact profile (OHIP-14), students, Croatia

## Abstract

Oral health-related quality of life (OHRQoL) is utilized in health services research to examine trends in oral health and population-based needs assessment. To determine the impact of oral diseases on everyday life, measures of oral quality of life are needed. In addition to common disease-based measures, they assess the need for oral care to evaluate oral health care programs and treatment management. The aim of this study was to evaluate the OHRQoL among Croatian university students. A cross-sectional study was conducted among 895 students (mean age 22 ± 4 years; 54.75% were females). The data collection was carried out through a self-administered short-form oral health impact profile (OHIP-14) questionnaire which comprises 14 items that describe 7 subscales. Each subscale is named according to its constitutive items: functional limitation, physical pain, psychological discomfort, physical disability, psychological disability, social disability and social handicap. The quality of data was descriptively analysed and internal consistency reliability was assessed by Chronbach’s alpha coefficient. Pearson’s correlation was performed on the OHIP-14 total score and 7 subscale scores. The level of significance was set to *p* < 0.05. The prevalence of reported impact on OHRQoL was 31.84% and the mean OHIP-14 score was 11.66 ± 8.72. Chronbach’s alpha for the OHIP-14 total score was 0.91 (range for subscales: 0.70–0.87). Total OHIP-14 score was correlated with each of the seven subscales (correlation range: 0.56–0.84). The psychological discomfort and physical pain subscales recorded the highest impact with 56.09% and 24.47%, respectively, while the least impact was recorded in the physical disability subscale with 13.35%. The mean OHIP-14 score of the students in this study reflects that the oral health status of most of the students did not significantly affect their OHRQoL. However, the psychological discomfort and physical pain subscales were the most severely affected aspects of their OHRQoL. The OHIP-14 had reasonable reliability in relation to subjective global oral health indicators among students and thus appears to be a useful OHRQoL measure in this context.

## 1. Introduction

Quality of life (QoL) has been the focus of many researchers for many years as one of the topics important in the life of every human being. Historically, definitions and measurements of QoL have varied and changed greatly [1]. QoL has become a subject of interest in psychology, philosophy, sociology, clinical medicine and dentistry, and in the health care system [2]. In the literature, there are numerous studies of QoL and certain aspects that constitute this quality. From the aspect of dentistry, the concept of oral health-related quality of life (OHRQoL) is particularly important. This concept has developed only in the last few decades, and it has not received much attention before [3]. QoL means personal satisfaction or dissatisfaction within the cultural or intellectual living conditions. The literature lists different approaches to this topic and many definitions of oral health and QoL, as well as many ways to assess this concept [4]. The World Health Organization (WHO) defines an individual’s QoL as the perception of their own position in life in the context of the cultural and value system in which the individual lives and in relation to their own goals, expectations, standards and values. It is a very broad term and is subject to the complex influence of physical health, psychological status, degree of independence, social relations, and personal attitudes toward common environmental characteristics [5]. There is a consensus that QoL is a multidimensional construct, and can be categorized into several dimensions such as emotional, physical, social and material well-being, and activity and development. The goal of modern dentistry is not only to improve oral health, but also to improve the overall QoL of patients. When assessing the outcome of a dental procedure, it is important to take into account the point of view of the dentist, but also that of the patient [6,7,8,9,10,11,12,13,14,15,16,17].

OHRQoL is defined as a multidimensional construct encompassing physical, social and psychological areas [18]. These aspects are not assessed by clinical examinations, only the presence and the severity of the disease are measured, thereby rarely taking into account the influence of the symptoms on QoL [6,10,19]. OHRQoL is evaluated by questionnaires that produce data on oral health and the effects of oral health on QoL. One of such questionnaires is the oral health impact profile (OHIP-14), which is used for the evaluation of the dimension of oral health effects on OHRQoL, such as functional limitations, physical pain, psychological discomfort, physical disability, psychological disability, social disability and social handicap [20,21].

The concept of health includes biopsychosocial well-being, and oral health can affect various aspects of QoL [22]. Despite the growing number of studies addressing this issue, few studies have included students as a specific population [23,24,25]. Such studies allow the analysis of OHRQoL among a group of people with intensive training in the detection of small deviations from normality, as well as in the way of maintaining oral health [26]. Numerous papers indicate changes in students’ attitudes and behavior regarding oral health as they progress in their studies [27,28,29,30,31,32,33,34]. Although this has not always been observed [35], students are expected to improve their health-related behaviors and attitudes by the end of the course [36]. The knowledge of students’ self-perceptions of oral health, including OHRQoL, will provide insights that can offer the definition of better teaching methods. Furthermore, understanding the OHRQoL can contribute to the development of strategies aimed at improving curricula and improving the health education of students. The aim of this study was to evaluate the OHRQoL among Croatian university students.

## 2. Participants and Methods

### 2.1. Participants

A cross-sectional study was conducted with university students (n = 928) in 2019, 903 of whom agreed to participate. However, 8 students (0.89%) were excluded due to incomplete questionnaires, so the final sample encompassed 895 students (99.11% response rate). All participants were generally healthy university students without oral conditions such as oral cancers, congenital craniofacial deformities, craniofacial trauma, etc. We also excluded students who were outside the target age (18–25 years), were not of Croatian nationality, had fixed orthodontic bands, or were pregnant. The study was carried out according to the Declaration of Helsinki. All students who agreed to participate signed a statement of informed consent.

### 2.2. Methods

#### 2.2.1. Assessment of Oral Health-Related Quality of Life

The short version of the oral health impact profile (OHIP-14) [20] was used for the assessment of OHRQoL. The period of reference was the previous 12 months and the questionnaire was self-administered by the students. The OHIP-14, one of the most commonly used generic OHRQoL measures, has proved reliable and valid among both young and middle-aged people in Sweden [37], Brazil [38], Scotland [39], New Zealand [40] and Japan [41]. The OHIP-14, containing psychometric properties and easily applied, is frequently used in the field of dentistry due to its solid conceptual and empirical foundation. It was validated for the Croatian population [24]. OHIP-14 answers to the questions are evaluated according to the Likert scale: 0—never, 1—hardly ever, 2—occasionally, 3—fairly often, 4—very often. The OHIP-14 score is calculated by adding up the responses on all 14 items, with an overall score ranging from 0 to 56. The higher the value of the results, the worse the OHRQoL [6,10,20,42,43,44].

A total of 50 undergraduate and graduate students were the participants of a pilot study with the aim of testing the methods and understanding the questions. The pilot study demonstrated no necessary changes to the methods, i.e., they proved adequate for the study on population.

The participants completed the questionnaires individually in a classroom, supervised by one of the researchers who addressed any issues that might have arisen. Anonymity and confidentiality were guaranteed. They were given the explanations of the objectives of the study. After the completion of the questionnaire the data collection process was carried out. The OHIP-14 questionnaire can be either self-administered or administered in interview form. However, the administration method does not affect the psychometric properties of the questionnaire [45].

The test-retest method was carried out on 10% of the sample in order evaluate the stability of the OHIP-14, with a two-month interval between separate administrations of the questionnaire. The weighted Kappa index demonstrated almost perfect agreement for the 14 items (0.92).

#### 2.2.2. Statistical Analysis

The collected research data were stored in a database in Microsoft Office Excel 2016 program and processed by a personal computer using the statistical program Statistica 13.1 (TIBCO Software, Palo Alto, CA, USA). The quality of data was descriptively analysed (mean value, standard deviation, median, minimum value, maximum value, and interquartile range) and internal consistency reliability was assessed by Chronbach’s alpha coefficient. Pearson’s correlation was performed on the OHIP-14 total score and 7 subscale scores. The variable OHRQoL was dichotomized as the absence of impact (answers of never and hardly ever) and the presence of impact (answers of occasionally, fairly often and very often). To determine the prevalence of impact on OHRQoL, for the total OHIP-14 and thus for the domains and single OHIP-14 items, the percentage of the presence of the impact (answers of occasionally, fairly often and very often) in relation to the total number of participants was calculated. The level of significance was set to p < 0.05.

## 3. Results

**Figure 1:**
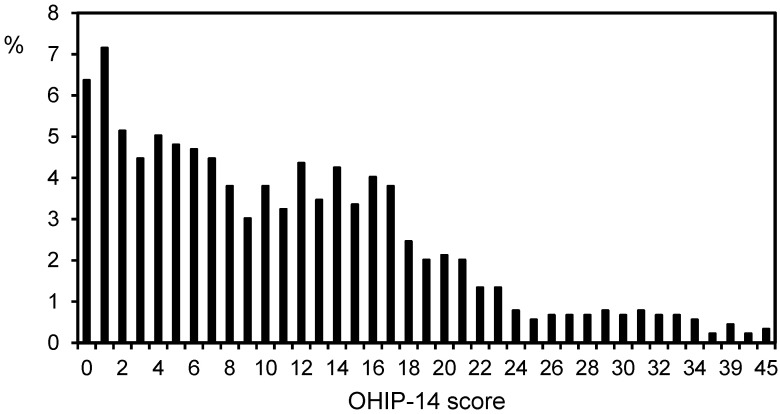
Histogram of the oral health impact profile (OHIP-14) score in the overall study sample (n = 895).

Mean age of the participants was 22 ± 4 years (54.75% were females). The prevalence of impact on OHRQoL was 31.84% and the mean OHIP-14 score was 11.66 ± 8.72 (range: 0 to 45). The highest mean scores were observed for the subscales psychological discomfort (3.45 ± 1.99), physical pain (1.85 ± 1.49) and psychological disability (1.66 ± 1.99), which were also the most frequently reported subscales with an impact on OHRQoL (56.09%, 24.47% and 24.25%, respectively). Those with the least impact were physical disability, social handicap and social disability (13.35%, 13.41% and 13.46%, respectively). Chronbach’s alpha for the OHIP-14 total score was 0.91 (range for subscales: 0.70–0.87). Total OHIP-14 score was correlated with each of the seven subscales (correlation range: 0.56–0.84) (Figure 1, Table 1). In relation to gender, no statistically significant differences were detected regarding the prevalence of the impact on OHRQoL for both the mean values of total OHIP-14 score and for individual OHIP-14 subscale scores.

With respect to single OHIP-14 items, the prevalence of OHRQoL varied from 9.61% (for the OHIP-14 item “totally unable to function because of problems with teeth, mouth or dentures” in the subscale social handicap) to 81.12% (for the OHIP-3 item “felt self-conscious because of problems with teeth, mouth or dentures” in the subscale psychological discomfort) (Table 2).

## 4. Discussion

Oral health is an integral part of general health and contributes to overall health QoL, while QoL issues are at the forefront of public health policy [46]. OHRQoL assessment allows the transition from traditional medical criteria to assessment and care that focus on social and emotional experience as well as a person’s physical functioning in defining appropriate goals and consequently the outcome of applied therapy and treatment procedures [10]. Subjective assessment of the patient contributes to making the right health decisions, and thus changes clinical practice and monitoring of the health outcomes of the treatment [47].

The mean OHIP-14 score recorded for students in the study was 11.66 ± 8.72. Since the mean value of OHIP-14 less than 14 indicates no impact on the oral health status of students [48], from the above it can be concluded that the impact of oral health status on daily life activities among students was of low intensity. Similar findings were recorded in the research conducted so far [23,31,48,49,50,51,52]. The important factors associated with a low OHIP-14 score are the low frequency and severity of oral problems and the inability of an individual to spot such problems [50]. Adverse oral conditions that have an impact on OHRQoL in this age group, such as periodontal disease or tooth loss, have a low incidence and severity of oral problems which may explain the low OHIP-14 score. The low frequency and severity of oral problems can also be influenced by oral health care in the surveyed students, given the expected fact that most of the participants in the study are well educated [26].

In this study, the subscales that contributed most to the impact on OHRQoL were psychological discomfort (56.09%), physical pain (24.47%) and psychological disability (24.25%), and one participant “felt self-conscious because of problems with teeth, mouth or dentures” (81.12%). Studies involving students in Russia, Pakistan, India, Brazil and Nigeria also report that psychological discomfort and physical pain have had the greatest impact on OHRQoL of students [23,24,48,50,52]. Psychological discomfort may be related to the level of concern students have about the appearance of their teeth and mouth, as they are encouraged to perceive and value their oral health status through the implementation of a health education curriculum. The subscale of physical disability recorded the smallest impact (13.35%) on the QoL of students, followed by the subscales of social handicap (13.41%) and social disability (13.46%). The low impact recorded for the social handicap subscale indicates that as a result of student’s oral health status they have not “felt that life in general was less satisfying” and that they are not “totally unable to function”. Also, the implementation of content from the domain of the health education curriculum contributes to ensuring emotional control of the individual to prevent psychological discomfort caused by oral health conditions from affecting their social relationships, which may explain the low impact of social handicap and social disability subscales on OHRQoL in our study. Although 56.09% of students reported that their oral health condition caused them considerable psychological discomfort, students did not allow this to significantly affect their social relationships, which may explain the low scores for the social disability and social handicap subscales. Similar findings have been reported in closely related studies [23,50,53]. During this study, only the OHIP-14 questionnaire was used, while visual and tactile methods of clinical dental examination were not included, which may lead to underestimation of dental caries. During further extensive research, the methods of clinical dental examination of students will be applied.

## 5. Conclusions

The mean OHIP-14 score recorded in this study reflects that the oral health status of most students did not significantly affect their OHRQoL. In this study, we found that the OHIP-14 subscales of psychological discomfort and physical pain were the most vulnerable aspects most affected by OHRQoL, which is consistent with most studies conducted so far in this age group. Therefore, it can be assumed that a similar pattern of OHRQoL exists in most young adults in different countries.

## Figures and Tables

**Table 1:** Descriptive statistics, internal consistency and correlation of OHIP-14 subscale scores and total score among Croatian university students (n = 895).

OHIP-14 Subscales	Score				Impact on OHRQoL		Cronbach’s Alpha	r
	Mean (SD)	Minimum	Maximum	Median (IQR)	No Impact (%)	Impact (%)		
Functional limitation	1.37 (1.36)	0	6	1 (0–2)	81.17	18.83	0.70	0.56 *
Physical pain	1.85 (1.49)	0	8	2 (1–3)	75.53	24.47	0.73	0.74 *
Psychological discomfort	3.45 (1.99)	0	8	3 (2–5)	43.91	56.09	0.75	0.78 *
Physical disability	1.15 (1.40)	0	7	1 (0–2)	86.65	13.35	0.71	0.76 *
Psychological disability	1.66 (1.99)	0	8	1 (0–3)	75.75	24.25	0.87	0.84 *
Social disability	1.11 (1.54)	0	8	0 (0–2)	86.54	13.46	0.80	0.82 *
Social handicap	1.07 (1.55)	0	8	0 (0–2)	86.59	13.41	0.72	0.83 *
OHIP-14 TOTAL	11.66 (8.72)	0	45	10 (5–17)	68.16	31.84	0.91	

OHIP-14: Oral Health Impact Profile; n: number of participants; SD: standard deviation; IQR: interquartile range; OHRQoL: oral health-related quality of life; r: Pearson’s correlation coefficient; *: statistically significant correlation on *p* < 0.05.

**Table 2:** Frequency of impact of each item of the OHIP-14 on OHRQoL among Croatian university students (n = 895).

Subscale/Item	0—Never		1—Hardly Ever		2—Occasionally		3—Fairly Often		4—Very Often	
	n	%	n	%	n	%	n	%	n	%
Functional limitation Have you had trouble pronouncing any words because of problems with your teeth, mouth or dentures?	652	72.85	155	17.32	69	7.71	16	1.79	3	0.33
Have you felt that your sense of taste has worsened because of problems with your teeth, mouth or dentures?	359	40.11	287	32.07	182	20.34	48	5.36	19	2.12
Physical pain Have you had painful aching in your mouth?	291	32.51	429	47.93	147	16.42	23	2.58	5	0.56
Have you found it uncomfortable to eat any foods because of problems with your teeth, mouth or dentures?	377	42.12	255	28.49	214	23.91	39	4.36	10	1.12
Psychological discomfort Have you felt self-conscious because of problems with your teeth, mouth or dentures?	83	9.27	86	9.61	294	32.85	263	29.39	169	18.88
Have you felt tense because of problems with your teeth, mouth or dentures?	371	41.45	246	27.49	175	19.55	59	6.59	44	4.92
Physical disability Has your diet been unsatisfactory because of problems with your teeth, mouth or dentures?	592	66.15	194	21.68	77	8.60	27	3.01	5	0.56
Have you had to interrupt meals because of problems with your teeth, mouth or dentures?	465	51.96	300	33.52	111	12.40	17	1.90	2	0.22
Psychological disability Have you found it difficult to relax because of problems with your teeth, mouth or dentures?	512	57.21	212	23.69	123	13.74	21	2.34	27	3.02
Have you been a bit embarrassed because of problems with your teeth, mouth or dentures?	419	46.82	213	23.80	179	20.00	46	5.13	38	4.25
Social disability Have you been a bit irritable with other people because of problems with your teeth, mouth or dentures?	543	60.67	217	24.25	101	11.28	16	1.79	18	2.01
Have you had difficulty doing your usual jobs because of problems with your teeth, mouth or dentures?	567	63.35	222	24.80	85	9.50	16	1.79	5	0.56
Social handicap Have you felt that life in general was less satisfying because of problems with your teeth, mouth or dentures?	572	63.91	169	18.88	96	10.73	36	4.02	22	2.46
Have you been totally unable to function because of problems with your teeth, mouth or dentures?	608	67.93	201	22.46	69	7.71	10	1.12	7	0.78

OHIP-14: Oral Health Impact Profile; OHRQoL: oral health-related quality of life; n: number of participants.

## Data Availability

The data presented in this study are available on request from the corresponding author.
